# Computational Models Describing Possible Mechanisms for Generation of Excessive Beta Oscillations in Parkinson’s Disease

**DOI:** 10.1371/journal.pcbi.1004609

**Published:** 2015-12-18

**Authors:** Alex Pavlides, S. John Hogan, Rafal Bogacz

**Affiliations:** 1 MRC Unit for Brain Network Dynamics, University of Oxford, Oxford, United Kingdom; 2 Nuffield Department of Clinical Neuroscience, John Radcliffe Hospital, University of Oxford, Oxford, United Kingdom; 3 Faculty of Engineering, University of Bristol, Bristol, United Kingdom; University Descartes, Centre National de la Recherche Scientifique, FRANCE

## Abstract

In Parkinson’s disease, an increase in beta oscillations within the basal ganglia nuclei has been shown to be associated with difficulty in movement initiation. An important role in the generation of these oscillations is thought to be played by the motor cortex and by a network composed of the subthalamic nucleus (STN) and the external segment of globus pallidus (GPe). Several alternative models have been proposed to describe the mechanisms for generation of the Parkinsonian beta oscillations. However, a recent experimental study of Tachibana and colleagues yielded results which are challenging for all published computational models of beta generation. That study investigated how the presence of beta oscillations in a primate model of Parkinson’s disease is affected by blocking different connections of the STN-GPe circuit. Due to a large number of experimental conditions, the study provides strong constraints that any mechanistic model of beta generation should satisfy. In this paper we present two models consistent with the data of Tachibana et al. The first model assumes that Parkinsonian beta oscillation are generated in the cortex and the STN-GPe circuits resonates at this frequency. The second model additionally assumes that the feedback from STN-GPe circuit to cortex is important for maintaining the oscillations in the network. Predictions are made about experimental evidence that is required to differentiate between the two models, both of which are able to reproduce firing rates, oscillation frequency and effects of lesions carried out by Tachibana and colleagues. Furthermore, an analysis of the models reveals how the amplitude and frequency of the generated oscillations depend on parameters.

## Author Summary

The collective firing of neurons is an essential mechanism for communicating information in the brain. However, in Parkinson’s disease there is a disruption of healthy brain dynamics, which interrupts the processing of normal movement. In particular, the neurons become overly synchronized and produce abnormally large oscillations in their activity. The frequency band of the oscillations associated with the difficulty of movement initiation in primate Parkinson’s disease is in the range of 13–30Hz, which is called the beta band. Understanding the generation of beta oscillations is important to improve treatments for Parkinson’s disease. Competing theories exist for how these oscillations are generated in the affected brain circuits, which include the motor cortex and a set of subcortical nuclei called the basal ganglia. This paper suggests two hypotheses for the generation of beta oscillations. The first hypothesis is that beta oscillations are generated in the motor cortex, and the basal ganglia resonate to the cortical input. The second hypothesis additionally proposes that feedback from the basal ganglia to cortex is critically important for the presence of the oscillations. We show that the models can successfully account for a wide range of experimental data concerning the presence of beta oscillations in Parkinson’s disease.

## Introduction

Excessive oscillations in the beta frequency range (13–30Hz) have been observed in the basal ganglia of patients with Parkinson’s disease [1]. The power of these oscillations is thought to be related to symptom severity because treatments that ameliorate symptoms, like dopaminergic medications and deep brain stimulation (DBS), also reduce the power of beta oscillations [2, 3]. Similar oscillations are seen in MPTP treated primates, albeit at a slightly lower frequency range of 8–15Hz [4].

Due to the complex architecture of the cortico-basal-ganglia-thalamic circuit, locating the origin of the pathological beta oscillations has been challenging, and several competing theories have been proposed. Below we briefly review the evidence suggesting involvement of the motor cortex, and a network composed of subthalamic nucleus (STN) and external segment of globus pallidus (GPe).

**Fig 1:**
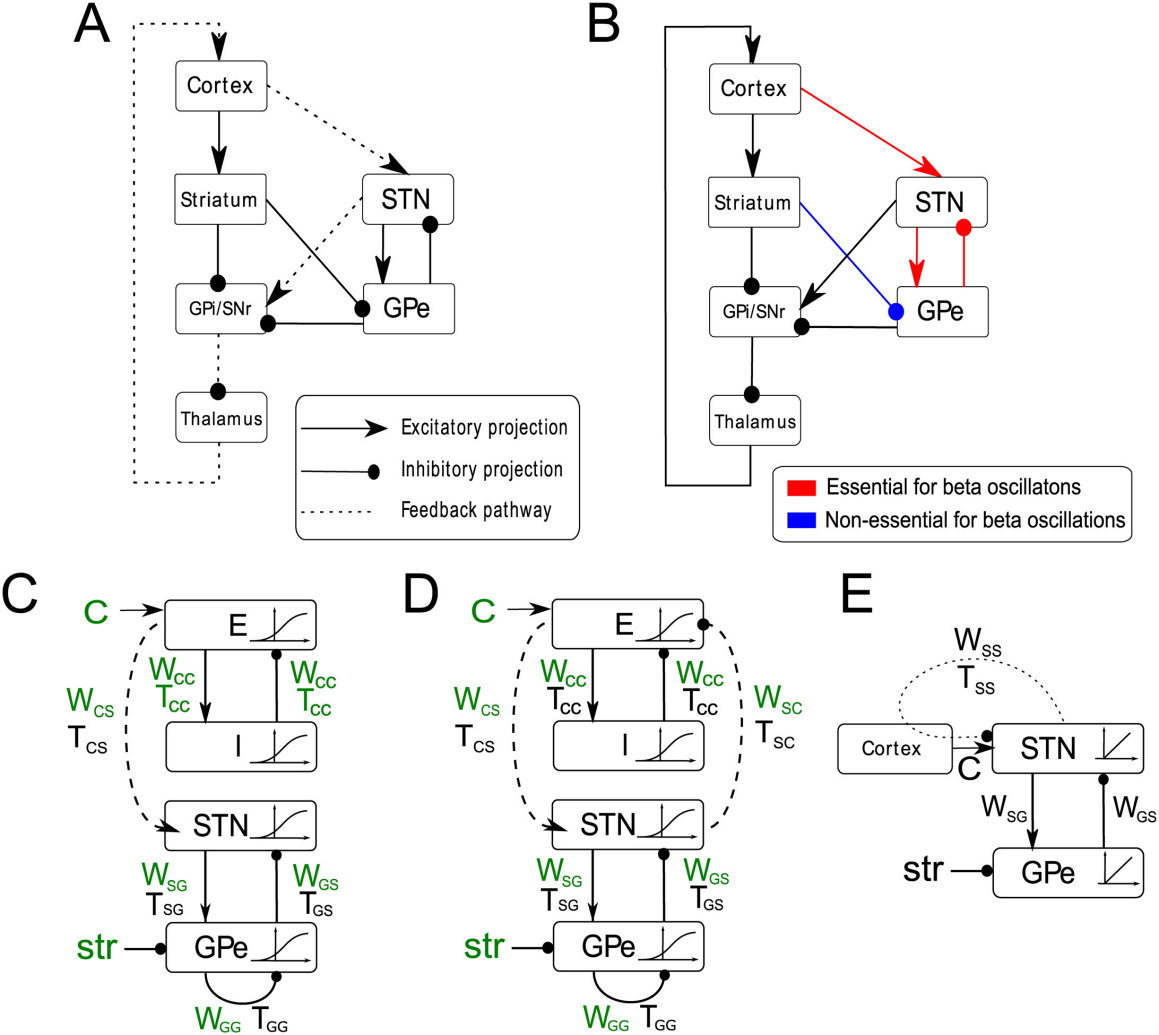
Connectivity in cortico-basal-ganglia-thalamic circuit and in the computational model. A) Major connections of the basal ganglia. Arrows denote excitatory connections and lines ending with circles denote inhibitory connections. The pathway which denotes cortical feedback via the hyperdirect pathway is highlighted with dashed lines. B) Summary of selected results of Tachibana et al. [14], who recorded activity of STN and GPe neurons in intact monkey model of Parkinson’s disease, and after blocking various inputs to the neurons in the vicinity of the recording electrode. Red lines indicate inputs that when blocked caused suppression of beta oscillations while the blue line indicates the striatal input to the GPe that when blocked did not reduce significantly the power of beta oscillations in the activity of recorded GPe neurons. C) A *resonance model* which includes time delays between the excitatory and inhibitory populations of the cortex, time delays between STN and GPe populations, time delays in the inhibitory GPe-GPe connections and further time delays connecting the cortex to the STN. This model also includes sigmoid activation functions for the STN, GPe, E and I populations which describe the input-output relationships of neurons in the populations. *w*_*ij*_ and *T*_*ij*_ denote the strength and delay of synaptic connections between neural populations *i* and *j*, where *i* and *j* can be equal to *C* for cortex, *S* for STN or *G* for GPe. Parameters shown in green are being fitted to the data. D) A *feedback model* that is identical to the model in panel C apart from including the time delayed connection between the STN and cortex, which forms a feedback loop between STN-GPe circuit and cortex. E) A linear model with time delay only in the cortical feedback.

It is thought that the STN-GPe circuit plays an important role in the generation of the pathological beta oscillations for several reasons [5]. Beta oscillations are prominent in the STN and GPe nuclei [6, 7], and they are effective targets of DBS [3, 8]. Neurons in the STN send excitatory connections to the GPe, while the GPe neurons send back inhibitory feedback to STN (Fig 1A), and theoretical work has shown that such architecture is prone to generating oscillations under certain conditions [9–13]. Critically, blocking the connections between the STN and the GPe abolishes the excessive beta oscillations [14].

It has also been proposed that the motor cortex plays an important role in the generation of pathological beta oscillation [3, 15–17]. It sends direct input to the STN, and receives indirect but prominent feedback from the STN and the GPe via the output nuclei of the basal ganglia and the thalamus (Fig 1A). In Parkinson’s disease increased coherence has been observed between beta oscillations in the cortex and the STN [3, 18, 19]. The causal role of the cortical input in producing excessive beta oscillations is suggested by observations that transcranial magnetic stimulation of the motor cortex reduces synchronized oscillatory neuronal activity in the STN-GPe circuit [20] as well as causing a significant reduction in akinetic and bradykinetic symptoms [21, 22]. This intervention may work by temporarily reducing oscillations in the cortex which would otherwise induce beta oscillations in the STN-GPe circuit, or by destabilising the rhythmic cortical feedback to the STN, via the hyperdirect pathway, which in turn would lessen the power of beta oscillations in the whole circuit [23].

Finally, data suggests that structures propagating activity from the STN-GPe circuit, including the internal segment of the globus pallidus (GPi) and the thalamus (Fig 1A), are also involved in transmission or generation of neural activity, resulting in symptoms of Parkinson’s disease. Lesions placed within this pathway in targets such as GPi and thalamus can bring about symptom improvements [24]. Moreover, DBS applied to GPi [25] or thalamus [26] can also reduce the symptoms. These interventions may work by disrupting the propagation of pathological beta oscillations from the STN-GPe circuit to brain-stem, or by reducing the feedback from STN-GPe network back to cortex.

Recently Tachibana et al. [14] presented data which are critical in helping to establish which connections in the cortico-basal-ganglia circuit are important in the generation of beta oscillations. These data provide strong constraints for a theory of beta oscillation generation in Parkinson’s disease and thus are of the central importance in this work. Tachibana et al. [14] investigated neural activity in a primate model of Parkinson’s disease not only in the intact animals, but also after blocking various inputs of the recorded neurons. They established that blocking the inputs indicated by red lines in Fig 1B abolished beta oscillations, while blocking the striatal input to the recorded GPe neurons, indicated by the blue line, did not. This data set provides many constraints which prove challenging to explain for existing mathematical models of beta oscillation generation. Because of its importance in motivating the new models, in the remainder of the Introduction we briefly discuss how the results of Tachibana et al. [14] relate to other theories of beta oscillation generation. Then we outline the main idea of the models that could be consistent with the results of Tachibana et al. [14], which we then present in more detail in the rest of the paper.

### Relationship of models to data from Tachibana et al

On the basis of published computational models, several theories for generation of beta oscillations have been formulated. McCarthy et al. [27] propose that beta oscillations originate in the striatum and spread to the rest of the circuit. On the basis of this theory one could expect the following three effects of manipulations of Tachibana et al. [14].

**Fig 2:**
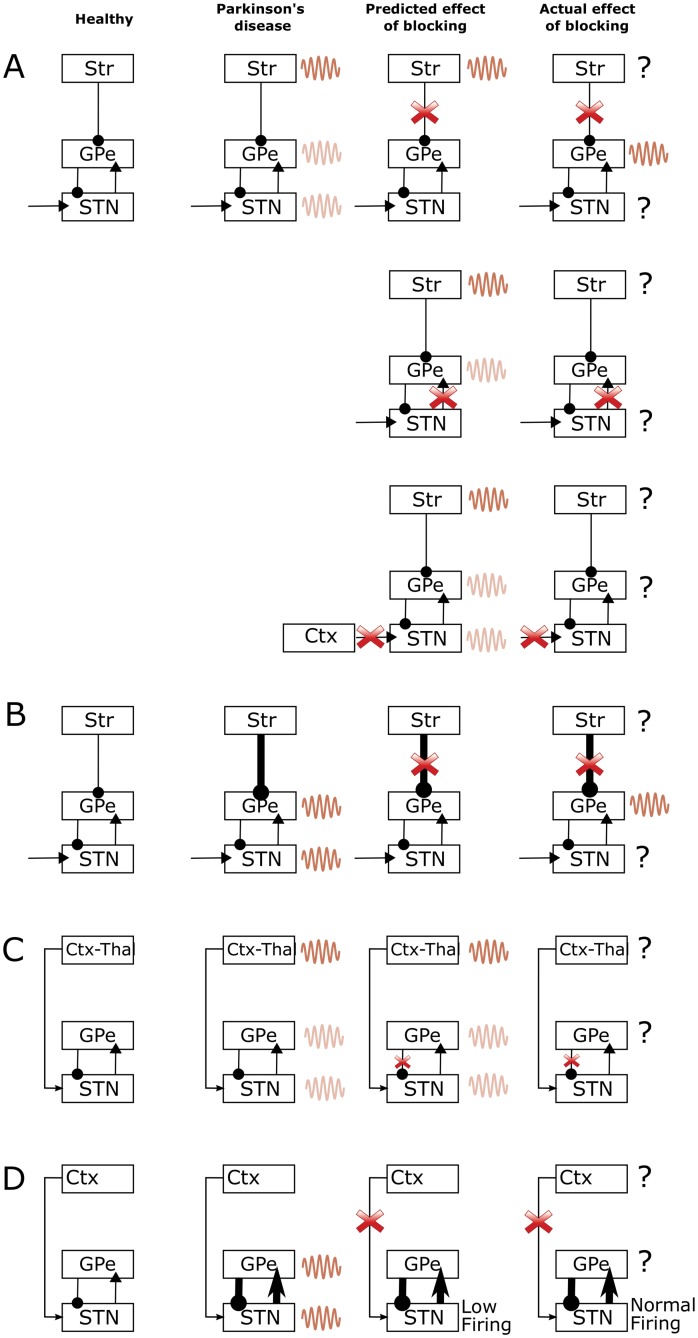
A graphical illustration of four alternative theories for the generation of beta oscillations in the cortical-basal ganglia circuits, the predicted effects of blockade from models and actual effects of blockade of connections reported in Tachibana et al. [14]. The first column shows the non-pathological state in healthy controls, the second column shows what the corresponding theories suggest happens in the Parkinsonian state, the third column shows the predicted effect of blockade of connection and the fourth column shows the actual effect of blocking from Tachibana et al. [14]. Note that oscillations in certain areas are not reported by Tachibana et al. [14], therefore in the fourth column this is illustrated by a question mark rather than oscillations in these regions. The dark coloured waves in the second and third column indicate the areas that generate the excessive oscillation according to a given theory, while the light coloured waves indicate the areas to which the oscillations spread. A) Model of McCarthy et al. [27] B) Model of Kumar et al. [10] C) Model of Van Albada et al. [31] and D) STN-GPe theory.

First, if beta oscillations were generated in the striatum, then one could expect that blocking the main routes from striatum to the rest of basal ganglia should reduce the power of beta oscillations. By contrast, Tachibana et al. [14] observed that blocking the striatal input to GPe and GPi in the vicinity of recording electrode did not reduce the power of beta oscillations in GPe, as illustrated in the top row of Fig 2A. An advocate of the striatal theory of beta generation could suggest that the reason why the power of beta oscillations was not reduced by blocking striatal input in the experiment of Tachibana et al. [14], is that they did not block all striatal inputs to GPe, but only the inputs in the vicinity of electrode. Thus beta oscillations could have propagated from striatum to other parts of GPe, then to STN, and then back to the part of GPe that was recorded.

Second, if the beta oscillations originated in the striatum, then one could also expect that blocking STN input to GPe should not abolish beta oscillations in GPe, as they should be directly provided to GPe from striatum. By contrast, Tachibana et al. [14] observed that blocking the STN input to GPe abolished beta oscillations in GPe, as illustrated in the middle row of Fig 2A. A possible explanation, in terms of the striatal theory, for the above observation is that blocking the excitatory STN input to GPe as carried out by Tachibana et al. [14] decreased responsiveness of GPe neurons. Therefore, because of this decreased responsiveness they did not transmit the input they received from striatum. Nevertheless, we note that although the firing rate of GPe neurons decreased after blocking glutamatergic transmission from STN, it was still relatively high (mean ∼30Hz according to Fig 9L in Tachibana et al. [14]). The f-I curves of GPe neurons (e.g. Figure 3B in Deister et al. [28]) suggest that GPe neurons are still very responsive to their input when firing at 30 Hz (i.e. 30 Hz is in a steep region of the f-I curve). Thus one could expect that if the GPe neurons firing at 30Hz received oscillatory input from the striatum, they should produce oscillations in the firing rate. An advocate of the striatal theory could suggest that another reason why blocking STN input to GPe abolished beta oscillations might have been that blocking STN input reduced the firing rate of arkypallidal GPe neurons projecting to striatum [29], which in turn disrupted generation of beta oscillations in the striatum.

Third, if the beta oscillations originated in the striatum, then one could also expect that blocking excitatory input to STN should not abolish beta oscillations in STN, as they should be propagated to STN from striatum via GPe. By contrast, Tachibana et al. [14] observed that blocking the excitatory input to STN abolished beta oscillations in STN, as illustrated in the bottom row of Fig 2A. It is noteworthy that blocking the excitatory input to STN resulted in a higher occurrence of bursting in the STN [14], suggesting that the STN neurons were more hyperpolarized, which led to the neurons producing after-hyperpolarizaton rebound responses and engaging in slower oscillatory rhythms.

Although one can propose an explanation for each inconsistency between the striatal theory and the Tachibana et al. [14] data, there are three observations that are different to what one could expect according to the striatal theory, while the models we will present later account for the data without the need for additional explanations.

Kumar et al. [10] showed that their model of STN-GPe circuit started to generate oscillations at beta frequency when GPe received increased input from the striatum, due to loss of dopaminergic modulation in the striatum. This result was also demonstrated and discussed previously by Gillies et al. [30]. These models predict that beta oscillations appear due to increased strength of the striatal input to GPe. Therefore, they predict that blocking striatal input to GPe should abolish beta oscillations, which is inconsistent with the data of Tachibana et al. [14] mentioned above, Fig 2B.

Van Albada et al. [31] proposed that the beta oscillations originate in cortico-thalamic loop and spread to the basal ganglia. If this were the case, then blocking connection from GPe to STN should not diminish the power of beta oscillations, as the STN would still receive direct input from cortex. By contrast, Tachibana et al. [14] observed that blocking the connections from the GPe to STN abolished beta oscillations, Fig 2C.

Finally, it has also been suggested that beta oscillations are generated internally within the STN-GPe network [11–13, 32–34]. In these models blocking cortical input would stop oscillations if this manipulation also reduced excitability in the STN-GPe network (Fig 2D). By contrast, Tachibana et al. [14] observed that after blocking the connections from cortex to STN, the beta oscillations were abolished even though the firing rate of STN neurons remained unchanged.

### Outline of the proposed models

Given the lack of a published model that could fully account for data of Tachibana et al. [14], we considered what theories could be consistent with it. First we considered if the data are consistent with a theory assuming that the beta oscillations are generated in the cortex, and that the STN-GPe circuits starts to resonate at beta frequency in Parkinson’s disease. Although this theory has been proposed before [5], we are not aware of any mathematical models which formalize it. Second, we considered a model which additionally assumes that the feedback from STN-GPe circuit back to cortex is necessary for maintenance of the oscillations.

To examine the plausibility of these hypotheses, we formalized them in simple computational models and investigated whether parameters values could be found for which the models reproduced the data on neural activity in primate model of Parkinson’s disease [14]. Given the large number of constraints, it was not obvious *a priori* whether the models would be able to fit the data. Below we show that the key features of the data set were successfully reproduced by both models. In order to differentiate between the two models, further experimental data is required, which we consider in the Discussion.

## Results

### Computational models

We developed minimal computational models, that can capture the hypotheses outlined in the Introduction. The architectures of the models corresponding to the two hypotheses are shown schematically in Fig 1C and 1D and we refer to them as resonance and feedback models respectively. The models include a circuit composed of interconnected neural populations in STN and GPe, which is based on an earlier model by Nevado-Holgado et al. [32]. In that study the STN-GPe circuit produced sustained or damped oscillations for certain ranges of parameters. Additionally, our models include a cortical circuit composed of excitatory and inhibitory neurons. In agreement with anatomical data, excitatory cortical neurons send projections to STN. In addition, there is a constant inhibitory input to GPe from striatum, as there was in earlier work by Nevado-Holgado et al. [32], and a constant excitatory input to the excitatory cortical population.

In the feedback model shown in Fig 1D we also included the projections from the STN back to cortex, which corresponds to a polysynaptic projection shown by dashed line in Fig 1A. Since this polysynaptic connection is formed by two excitatory and one inhibitory connections, its overall effect is inhibitory. Such a feedback connection is not present in the resonance model (Fig 1C), which was the only difference between the models.

The models describe the average instantaneous firing rate in the STN and GPe (rather than the activity of individual neurons), which results in a relatively small number of parameters. The values of most parameters were available from published studies. We investigated if we can find realistic values of the remaining parameters (describing strengths of synaptic connections between different neural populations) for which the models reproduce patterns of activity in a monkey model of Parkinson’s disease, observed by Tachibana et al. [14]. To search for such parameters we constructed a cost function which described by how much the firing rate, the amplitude and frequency in simulations differed from those observed by Tachibana et al. [14]. The cost function also included terms that increased its value if the effects of blocking various connections differed from those observed by Tachibana et al. Search for parameters minimizing this cost function was run 200 times for both the resonance and the feedback model, to determine if the resulting sets of parameters were similar or if they formed distinct clusters in the parameter space.

### Models reproduce key aspects of experimental data

**Fig 3:**
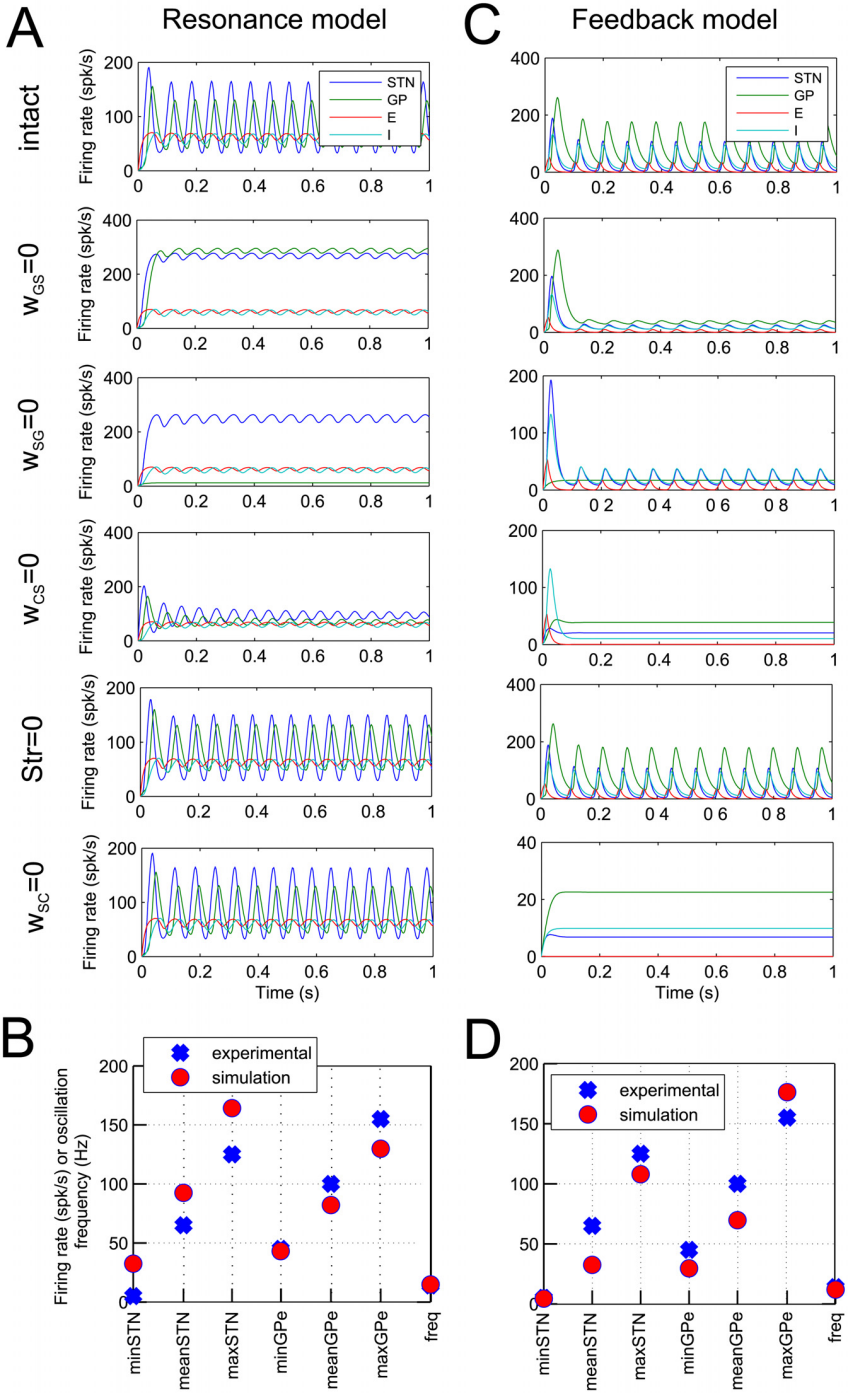
Results of simulations of the resonance model (panels A and B) and feedback model (panels C and D). A, C) Each of the six panels shows the activity of the STN, GPe and cortical populations as a function of time. The labels to the left indicate if a row shows simulations of an intact model, or a model with particular connections blocked. In simulations in panel A the following parameters were used: *w*_*SG*_ = 4.87, *w*_*GS*_ = 1.33, *w*_*CS*_ = 9.98, *w*_*SC*_ = 8.93, *w*_*GG*_ = 0.53, *w*_*CC*_ = 6.17, *C* = 172.18, *Str* = 8.46, *T*_*CC*_ = 4.65, *τ*_*E*_ = 11.59, *τ*_*I*_ = 13.02, *B*_*E*_ = 17.85, *B*_*I*_ = 9.87, *M*_*E*_ = 75.77 and *M*_*I*_ = 205.72. In simulations in panel C the following values were used: *w*_*SG*_ = 2.56, *w*_*GS*_ = 3.22, *w*_*CS*_ = 6.60, *w*_*SC*_ = 0.00, *w*_*GG*_ = 0.90, *w*_*CC*_ = 3.08, *C* = 277.94, *Str* = 40.51, *T*_*CC*_ = 7.74, *τ*_*E*_ = 11.69, *τ*_*I*_ = 10.45, *B*_*E*_ = 3.62, *B*_*I*_ = 7.18, *M*_*E*_ = 71.77 and *M*_*I*_ = 276.39. B, D) The comparison between experimental and simulated statistics of the oscillations.

Fig 3 presents simulations of resonance and feedback models with best parameter sets. The top displays in Fig 3A and 3C show the activity of simulated neural populations in the STN and GPe. The models successfully reproduce the minimum, mean and maximum firing rates and the oscillation frequency of the sample neurons in the STN and GPe, which are compared in Fig 3B and 3D. The other displays in Fig 3A and 3C show the activity in the models after blocking different connections, as indicated on the left to the displays. In agreement with the observations of Tachibana et al. [14], the beta oscillations are significantly attenuated in the model by blockade of the STN-GPe or the GPe-STN connections, or by blockade of input to the STN from motor cortex. In case of the resonance model, firing rates in STN and GPe after lesion of *w*_*SG*_ or *w*_*GS*_ are higher than would be expected. This is because there was no term included in the cost function to constrain firing rates after lesion of these connections. It is possible that in the biological system there are compensatory or homeostatic mechanisms within the STN and GPe that maintain levels of activity, which are not included in our model.

In addition, blocking striatal input increased the mean firing rate of the GPe population while leaving the power of the oscillations relatively unchanged, which agrees with the data from Tachibana et al. [14]. The frequency of oscillations produced by the models are 15*Hz* and 12*Hz*, which compares favourably with the beta oscillations recorded in primates [4].

Finally, the bottom panels in Fig 3A and 3C show activity after blocking the STN feedback to cortex. Such feedback is not present in the resonance model, so this panel looks identical as the simulation of the intact model, and is just included for comparison with the feedback model. In the simulation of feedback model, blocking the feedback from STN to cortex does stop the oscillations, so the two models will make different predictions with respect of experimental manipulations that can correspond to blocking this connection and we come back to this issue in the Discussion.

### Parameters of the models

Since in neural circuits, the oscillations are generated by neural populations connected in loops, parameters which particularly influence the stability of the model are the ones which describe the coupling in the main loops of the two models (see Fig 1C and 1D): the STN-GPe loop (*w*_*SG*_, *w*_*GS*_), the loop within the cortex (*w*_*CC*_), and the long feedback loop (present only in Fig 1D) connecting the two loops (*w*_*CS*_, *w*_*SC*_), to which we will refer as the *long loop*. As two of these loops are described by pairs of parameters, in analyses below we summarize each pair by their geometric mean. Hence we obtain 3 values ($\sqrt{w_{SG}w_{GS}}$, *w*_*CC*_, $\sqrt{w_{CS}w_{SC}}$), which summarize the coupling in these 3 loops.

**Fig 4:**
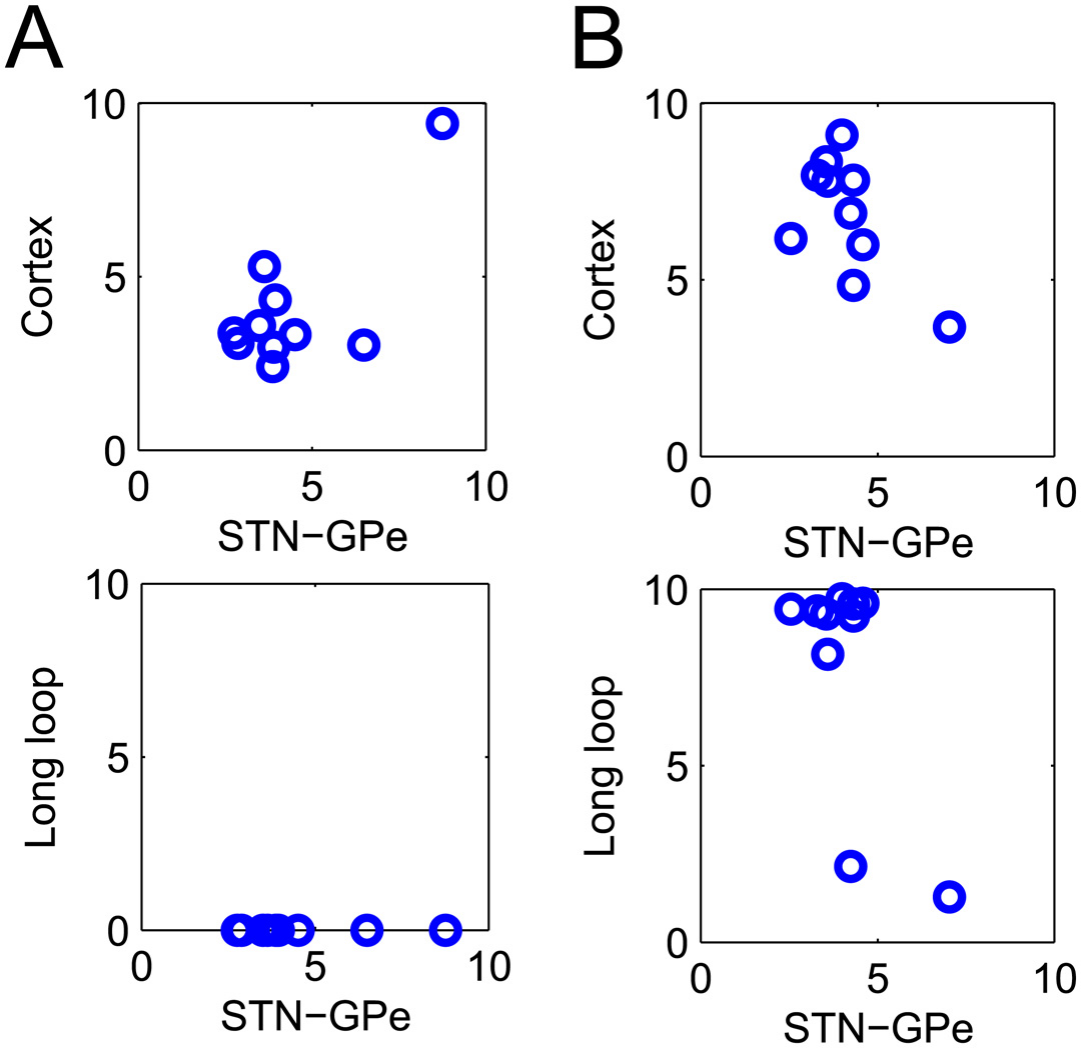
The top ten parameter sets found by the optimisation procedure. Of interest are the parameter values that represent the STN-GPe circuit, the cortex and the closed basal ganglia-cortical loop. The relationship between these values are shown in the two panels, where each circle represents a solution of the optimisation. A) Resonance model. B) Feedback model.

The coupling in the loops in the top ten parameter sets found by optimisation are shown in Fig 4A and 4B for the resonance and feedback model, respectively. Each circle in the figure represents the parameter values for that particular parameter set. Two panels are shown, which visualize the relative strength of the STN-GPe vs. the cortical loop and the STN-GPe loop vs. the long loop.

All parameter sets found for the resonance model shown in Fig 4A suggest that a strong cortical oscillation is required to support beta oscillations in the STN-GPe circuit. The long loop values are zero, because *w*_*SC*_ = 0 is assumed in the model, and they are just shown for comparison with the feedback model.

The parameter sets found for the feedback model are shown in Fig 4B. The bottom panel shows two clusters of parameter sets: the first with a strong long loop and the second with a weaker long loop. The behaviour of the model for one of the parameter sets from the first cluster is shown in Fig 3C. For the parameter sets in this cluster, the long loop is critical for oscillations, and blocking it stops oscillations (bottom panel in Fig 3C).

In the second group of parameter sets, the behaviour of the model is rather similar to that shown in Fig 3A for the resonance model. In particular for these parameter sets the cortical feedback is not critical for oscillations, as setting *w*_*SC*_ = 0 does not reduce the oscillations. It is not surprising that some of the parameter sets found for the feedback model have a weak long loop, as the resonance model with *w*_*SC*_ = 0 can also reproduce the key aspects of the data.

### The dependence of oscillations on connection weights

For a particular set of model parameters we have shown above that blocking connections within the STN-GPe network or within the long feedback loop can lead to the attenuation of beta oscillations. However, since these parameter values were estimated by fitting the model to the data from a sample of STN and GPe neurons, uncertainty exists about these values, and hence it is important to characterize how the presence of oscillations depends on strength of synaptic connections in general.

Modifying the strengths of synaptic connections in the feedback model will change its stability by changing two factors: (i) the coupling between neural populations, and (ii) the level of excitability in different neural populations. To illustrate the second factor, consider an example of increase in connection from GPe to STN. Although it increases the coupling between STN and GPe, it may stop oscillations simply because the STN neurons may receive too much inhibition and may be unable to fire. Mathematically, this would occur because of the non-linear shape of function *F*_*S*_: If the overall input *x* to STN become so negative that function *F*_*S*_(*x*) is flat in the range of *x* (recall that we assumed functions *F* to be sigmoidal, and sigmoidal function are flat for very small and and very large inputs), then the activity in STN will not change due to changes in its input, which prevents STN from contributing to oscillations.

To avoid this complication, and focus on the effect of coupling between neural populations, we analysed the behaviour of the linear version of the model in which we set the functions *F* to *F*(*x*) = *x*. We used the parameters of the model corresponding to Fig 3C, except for the parameters controlling the coupling in the three loops: *w*_*SG*_, *w*_*GS*_, *w*_*CC*_, *w*_*CS*_, *w*_*SC*_, which we varied systematically between simulations. Furthermore, we set *w*_*SG*_ = *w*_*GS*_ and *w*_*CS*_ = *w*_*SC*_, so we really varied 3 independent parameters describing the coupling in the 3 loops.

**Fig 5:**
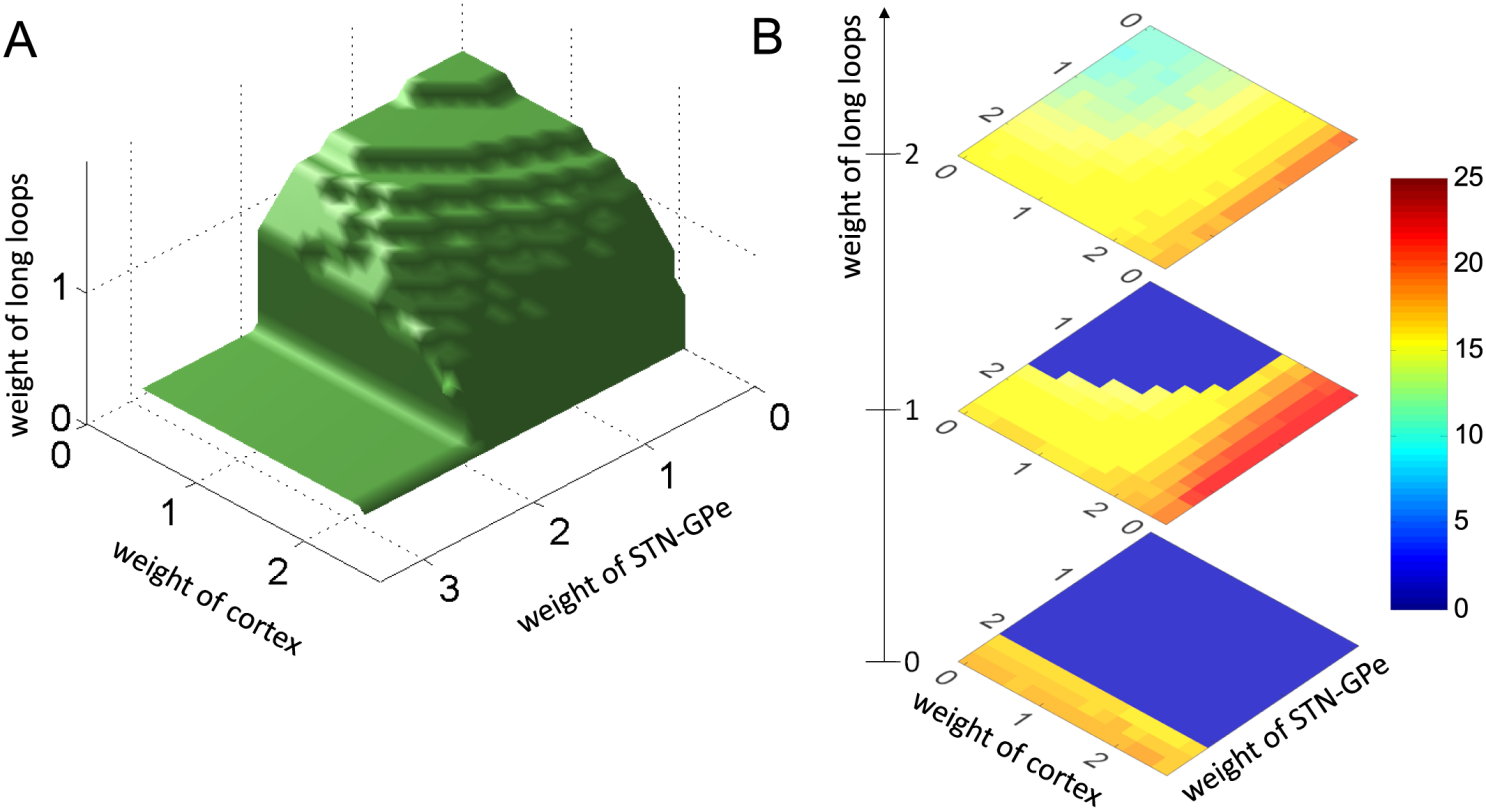
The dependence of oscillations on connection weights. A) Stability of the model for different strengths of connections in the model. The model displays non-oscillatory behaviour when the parameters are below the surface and oscillatory behaviour when parameters are above the surface. B) The frequency of oscillations for different strengths of connections in the model. The three displays show frequency of oscillations for three values of *w*_*CS*_ (indicated next to the number line). Dark blue corresponds to parameters for which no oscillation are produced.

The stability was assessed by varying the strength of the three loops and finding the regions in the parameter space where the STN activity was stable. Fig 5A shows the boundary of this region. Thus the system is stable for parameters below the surface, and produces oscillations above it.

Fig 5A shows that by increasing any of the 3 parameters while keeping others equal to 0 can cause oscillations, so each of the loops can generate oscillations on its own. For example when *w*_*CC*_ = *w*_*SC*_ = 0, which corresponds to the situation where STN does not receive any periodic input, the model can produce oscillations [32], when the connections within the STN-GPe network are sufficiently strong. Similarly increasing the feedback in the long loop while keeping *w*_*CC*_ = *w*_*SG*_ = 0 can produce oscillations. Finally, increasing coupling in the cortex will produce oscillations there, but for these oscillations to be visible in the STN, they need to propagate to STN via the long loop, hence the weights of long loop need to be above 0.

The value of the weights for which the loops generate the oscillations on their own differ between loops. It is lowest for the long loop (around 1.6) as it has longest delays, which makes this loop least stable on its own.

Importantly, Fig 5A illustrates that there are many combinations of parameter values for which the system is unstable and yet none of the loops can generate oscillations on its own (these are the points above the surface but for which *w*_*CS*_ < 1.6, *w*_*CC*_ < 2, *w*_*SG*_ < 2). In such cases the oscillations are not generated in any single loop but come from interaction among them. For these parameters blocking connections within any loop may stop beta oscillations.

Fig 5B show the how the frequency of the oscillations depends on the connection weights. First, we note that the oscillations produced by different connection strengths are relatively similar, and all fall close to the beta range. This similarity is due to the fact that in all simulations the values of transmission delays and time constant were the same, and these parameters rather than connection weight mainly determine the frequency of oscillations (as has been shown in [32] for the model of STN-GPe circuit). Second, we note that the actual value of the frequency depends on which loop mostly contributes to the oscillations: The frequency is lowest, when oscillations are driven by the long loop (corresponding to high weight in the long loop and low weights in other loops), because the long loop has longest delays. By contrast the frequency is highest, when the oscillations are mostly driven by cortical loop, because the cortical loop has relatively short delays between excitatory and inhibitory neurons and these neurons have fastest time constants in the model.

### Dependence of oscillation frequency on the delay in cortical feedback

**Fig 6:**
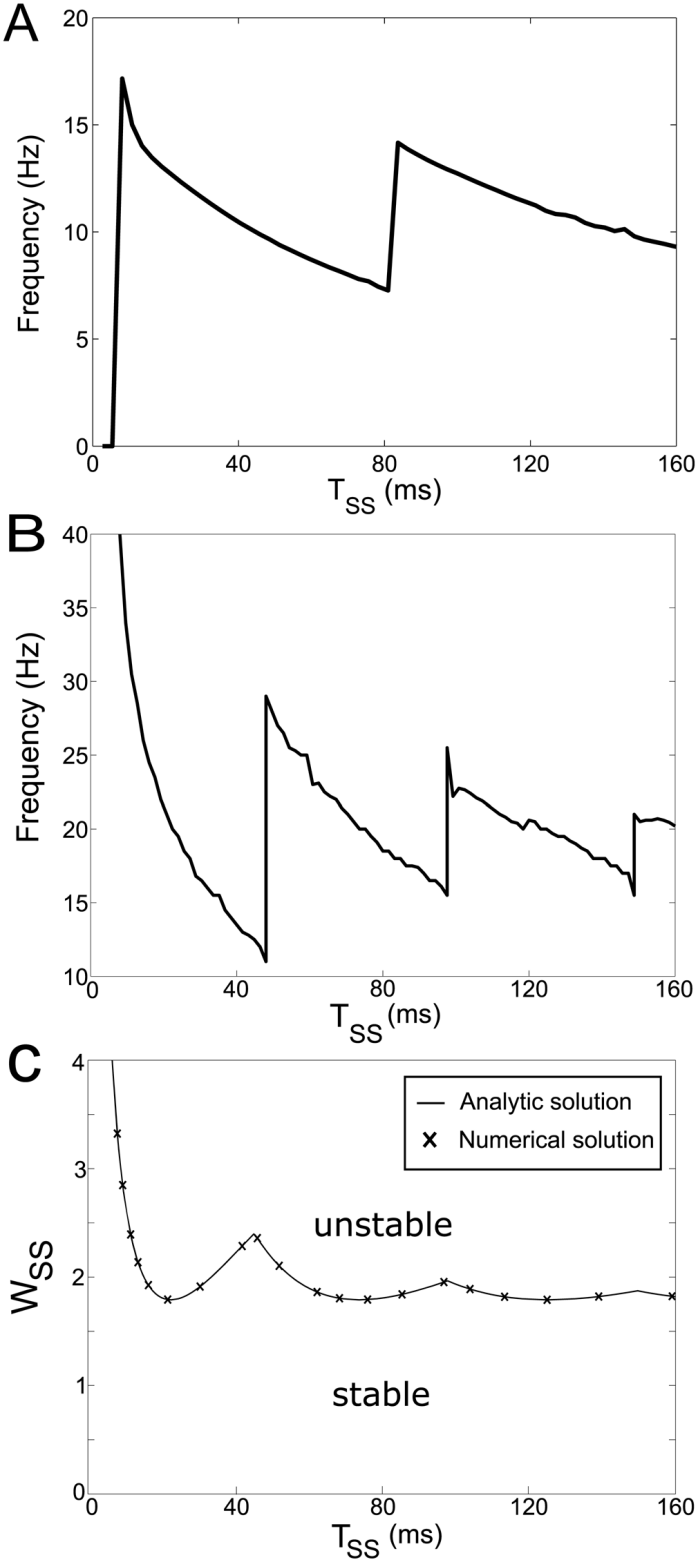
Effects of time delay in cortical feedback on model behaviour. A) The dependence of frequency in the feedback model on the delay in the long loop. B) The frequency range of oscillations produced by the linear system with delay only in cortical feedback for parameters that correspond to those along the stability boundary in panel C (i.e. for *w*_SS_ slightly higher than the one at the stability boundary for a given *T*_SS_). C) The stability boundary of the linear system with delay only in cortical feedback. The analytic solution (solid curve) is overlaid with the numerical solutions (crosses). When the system is in the parameter region above the stability boundary the STN-GPe circuit oscillates.

Fig 6A show the dependence of oscillation frequency in the feedback model on the time delay in the long loop. Interestingly, this relationship has a non-monotonic and periodic structure.

To understand the effect in detail, we analysed a simplified version of the model, which is necessary to solve the model analytically. In particular, we followed the approach of Dovzhenok and Rubchinsky [35] and instead of explicitly modelling the activity in all neural populations in the feedback loop, which connect the STN to the cortex and cortex to STN (indicated by dashed lines in Fig 1A), we captured it in a single connection with time delay. In addition, the simplified model is linear and excludes time delays in the STN-GPe circuit (without the delays, the STN-GPe network in the model cannot generate oscillations on its own [32], so any oscillations that are produced arise from interactions between STN-GPe circuit and the long loop). Furthermore, the simplified model excludes the self-inhibition of the GPe population (*w*_*GG*_ = 0). For more details see section Models below. The simplification of the network is shown schematically in Fig 1E. The frequency of oscillations produced by the simplified model is shown in Fig 6B. It highlights that the frequency produced is a non-monotonic function of the feedback time delay.

To understand this non-monotonic relationship intuitively, it is useful to consider a mechanical analogy. In the model with parameters used in Fig 3C, the STN-GPe network has tendency to resonate at beta frequencies, but on its own is unable to sustain these oscillations, thus it could be compared to a playground swing with a young child who is not yet old enough to move the swing alone. The cortex provides feedback that depends on past activity, so it could be compared to a father providing a force to the swing that is proportional to its past position, which can keep the swing moving. The father’s force will speed up the oscillations of the swing for the time delays for which the force coincides with direction of movement of the swing, while for other delays, the feedback may slow down the frequency of the swing. If the delay of father’s feedback is increased by a period of the swing, the effect of feedback will not change much, which gives the repetitive structure seen in Fig 6A.

The system is stable in the region below the curve in Fig 6C, where crosses show the stability boundary obtained by numerical methods using DDE-BIFTOOL, which agrees with the analytic boundary. To observe the non-monotonic relationship between frequency and the delay time, the system must first be in the unstable parameter region of Fig 6C where oscillations occur. One can observe in Fig 6C that for certain strengths of cortical feedback, e.g. *w*_SS_ = 2, the presence of oscillations depends periodically on the feedback time delay. This could be understood by considering the mechanical analogy of a father pushing a swing with a force proportional to the position of the swing with certain time delay. The length of this time delay will determine if the father’s force will tend to amplify or dampen the oscillations of the swing.

As described in the section *Models* this model is mathematically equivalent to a system for which the stability has been previously analysed by Cooke and Grossman [36].

## Discussion

We have demonstrated that the model of the STN-GPe circuit receiving input from a cortical oscillator can reproduce both the oscillations at beta frequency observed in the sample STN and GPe neurons and the effects of blocking different connections in 1-methyl-4-phenyl-1,2,3,6-tetrahydropyridine-treated Parkinsonian monkeys [14]. Furthermore, it was also shown that a model that also includes feedback from STN back to cortex, can also reproduce the key aspects of data of Tachibana et al. [14]. An analysis of the model shows that stronger coupling in any of the three loops included in the model (STN-GPe circuit, cortex, long loop) leads to greater instability of the system, and may provide a significant contribution to the generation of beta oscillations. This suggests a possibility that beta oscillations could be an emergent property of the entire cortico-basal ganglia networks, rather than a localised phenomenon that spreads throughout the circuits. This helps to explain why interactions in different nuclei within this closed cortico-basal ganglia loops can influence the power of beta oscillations throughout the circuits and the symptoms of Parkinson’s disease.

### Experimental predictions

There were two parameter regimes for which the model was able to account for the data: In the resonance model, or some estimated parameter sets of the feedback model, the weight of the connection from STN back to cortex was equal or close to zero, thus it did not contribute to generation of oscillations in the model. By contrast in most parameter sets of the feedback model, this connection was critical for the generation of oscillations. In these two parameter regimes blocking feedback from STN to cortex either did not or did stop the oscillations—compare bottom panels of Fig 3A and 3C.

To our knowledge, no data is available on whether lesion of GPi or thalamus reduce beta oscillations in the STN. To establish whether the feedback connection from STN to cortex is critical for generation of beta oscillations, one could test if lesioning or inactivation of the GPi or the thalamus reduces the power of beta oscillations in the STN.

### Relationship to other models

The presented models can be viewed as extensions of previous models describing STN-GPe circuit. These previous models are very useful for understanding the contribution of this circuit to beta oscillations, but in this paper we point out that other loops and connections in the cortico-basal-ganglia network are also likely to contribute.

Most models discussed in the Introduction and the models presented in this paper explicitly encapsulate a particular theory on the origin of beta oscillation. An alternative approach was taken by Marreiros et al. [37] who constrained a statistical model describing dynamics in the cortico-basal-ganglia circuit by data collected from Parkinson’s disease patients, using dynamic causal modelling (their model does not generate oscillations on its own, but it is rather a statistical model that can be fit to data). Their findings are in broad agreement with our model, suggesting that the STN-GPe circuit is essential for pathological oscillations and that input from the cortex is required to maintain these oscillations. They find that partial or full lesions in their model of reciprocal connections in the STN-GPe loop ameliorate beta oscillations and that in addition the full, but not partial lesions, of the hyperdirect input to STN, or the connection from STN to GPi, also stop pathological oscillations.

### Changes leading to development of pathological oscillations

The analysis of our models suggests that the beta oscillations may start to emerge when connections become stronger in either the STN-GPe loop, cortex, or the STN-GPi-thalamus-cortex-STN loop.

Previous modelling studies [32, 38] have already proposed that the oscillations in Parkinson’s disease emerge due to an increase in connection strengths within STN-GPe network. The increase in the STN-GPe connectivity is supported by observations [39, 40] that both the STN and GPe contain D2 receptors (and dopamine has an inhibitory effect on synapses containing D2 receptors). More direct evidence for increased connections from the GPe to STN in Parkinson’s disease is provided by observations that dopamine depletion increases the number of synaptic connections between the GPe and STN [41] and the inhibitory currents in the STN neurons due to activating GABA receptors [42].

In addition, it is likely that the connections in the STN-GPi-thalamus-cortex-STN loop also become stronger in Parkinson’s disease. The increase in effective connectivity from the cortex to the STN is suggested by an analysis of local field potentials with dynamic causal modelling [43]. Furthermore, an increase in excitatory currents in the STN neurons due to activating AMPA receptors [42] may suggest that the STN responds more to rapid changes in cortical input in Parkinson’s disease.

### Interpretation of Tachibana et al. data

In the Introduction we highlighted that the experiment of Tachibana et al. [14] provides extremely useful constraints that may distinguish between different theories of generation of the pathological beta oscillation. Therefore, we feel it is important to investigate how the findings of Tachibana et al. [14] generalize to other species and manipulations. For example, it is important to test if pathological beta oscillations would be abolished when striatal input to all neurons in GPe were blocked, because different models make different predictions on the effect of this blockade.

Tachibana et al. [14] demonstrated that blocking glutamatergic inputs to the STN stops beta oscillations in the STN. We have assumed, as did Tachibana et al. [14], that this is critically dependent on a reduction in the input from cortex, via hyperdirect pathway. However, inputs from the centromedian–parafascicular thalamic complex (CM–Pf) [44] and pedunculopontine nucleus also project excitatory glutamatergic projections to the STN, which are also blocked, and may therefore contribute to this reduction. While these other inputs cannot be excluded outright there are good reasons to consider the excitatory inputs via the hyperdirect pathway as being a very important driver of beta oscillations in the STN. In particular, beta activity in cortex has been shown to be correlated with beta activity in the STN [7, 19, 45, 46].

### Species variation in pathological beta frequency

The feedback model provides a possible insight on the non-monotonic dependence of pathological beta frequency on the brain size, as monkeys tend to have a lower frequency of pathological oscillations than humans and rats [3, 18]. These results seem surprising because monkey and human physiology and size are closer to one another than either of them are to the physiology of rats. The size of the animal is likely to affect the time delay in the transmission around the STN-GPi-thalamus-cortex-STN loop. The analysis of the model (Fig 6A–6B) has shown that increasing this time delay can first decrease and then increase the frequency of oscillations. We are not explaining quantitatively the difference in frequency between species, as species differ in more aspects and the model has been predominantly constrained by primate data, but we illustrate that the brain size can have counter-intuitive effects on the frequency of generated oscillations.

### Limitations of the model and future direction

The models presented in this paper make many simplifying assumptions, the validity of which needs to be verified in future work. The first of which concerns whether a mean field model is appropriate for the study of beta oscillations in the basal ganglia circuits. We believe that a mean field model is one method, amongst others, that is appropriate for the investigation of beta oscillations in the cortico-basal ganglia circuits. In particular, there is a high level of neural redundancy due to the spatial ubiquity of beta oscillations throughout these circuits. This large scale spatial-temporal activity was one of the motivating factors that led Wilson and Cowan [47] to develop their model of excitatory and inhibitory interactions in localised populations of neurons, and therefore its application to this problem is a natural choice. Furthermore, because a mean field model describes the large scale properties of cells and the relative connectivity between populations of cells, the analysis (which involves relatively few parameters) is more tractable and has more clearly interpretable results.

A further simplification concerns the use of time delayed variables, which are used in models to include events whose underlying dynamics either cannot be precisely observed or to simplify complex processes by abstraction. A time delay typically represents the time necessary for the underlying network of events to produce some result observable in the higher level model [48]. By simplifying much of the neural connectivity in our model using time delayed variables we were able to investigate a number of questions related to the generation of beta oscillations in the cortico-basal ganglia circuits. A similar approach was promoted by Dovzhenok and Rubchinsky [35], in a model of tremor oscillations in the same cortico-basal ganglia circuits. They state that the limitations of this modelling approach is both its advantage (since it allows us to study the generic effects of feedback) and its disadvantage (since there are many limitations and open questions related to it). In particular, the STN-GPi-thalamus-cortex feedback loop is a simplification of a much more complicated system, with many processes that occur between the output nuclei of the basal ganglia and cortex. Furthermore, our simulation of the interruption of this loop was simplified. Since the loop contains both excitatory and inhibitory nodes, depending on where the loop is cut, it may excite or inhibit the cortex. For example, if the GPi is lesioned, this will dis-inhibit the thalamus and potentially excite the cortex. However, if the thalamus is lesioned this may cause the activity of the cortex to decrease because it no longer receives excitation from the thalamus. Little is understood about how lesions of nuclei in the basal ganglia change activity in the cortex. In particular there may be a number of compensatory mechanisms that occur after lesions that will influence cortical activity. This is an area of research that needs development.

For simplicity, the current models only describe the average activity of all neurons in GPe. However, it has been demonstrated that there exist two separate populations of GPe neurons that respond in different phases of beta oscillations [6], and project to different structures within the basal ganglia [29]. These populations were included in a recent model describing the propagation of beta oscillations [49], and it would be interesting to include them in a model describing generation of beta oscillations.

It has been shown directly [14] and indirectly [50] that blocking of the hyperdirect pathway does not reduce the mean firing rate of STN neurons. In the model we include a constant excitatory input to cortex to reflect this. However, an explanation for why the mean firing rate of STN neurons remains constant after blockade of hyperdirect pathway remains unexplained and warrants further research. It is possible that this behaviour is related with increased bursting reported by Tachibana et al. [14] after blocking glutamatergic input to STN. After the blocking the STN neurons would be hyperpolarized, and the STN neurons are known to produce burst when hyperpolarized due to slow calcium currents [51–53]. Alternatively, another source of continued firing could be due to the activation of metabotropic glutamate receptors in the STN, which were not blocked by Tachibana et al. [14]. Ionotropic glutamate receptors, which were blocked by Tachibana et al. [14], are responsible for fast excitatory transmission within the central nervous system. Since they act directly on ion channels of the post-synaptic membrane these receptors are likely to carry the major cortical effect on STN neurons. Conversely, metabotropic receptors are slower acting and have diverse modulatory effects on glutamate transmission, rather than acting directly on ion channels [54, 55]. While it is apparent that blocking ionotropic receptors lead to amelioration of beta oscillations in the STN, more research is needed to answer whether continued activation of metabotropic receptors could sustain firing rates of STN neurons. Future insight into this could help to refine the model.

Finally, the models suggest that beta oscillations may develop due to strengthening the connections and feedback between various neuronal populations. It would be interesting to model how and why these connections get stronger in dopamine depletion.

## Models

### Description of the model

We consider models describing the generation of beta oscillations in the cortco-STN-GPe network, that can accurately reproduce observed patterns of neural activity, but which at the same time are as simple as possible, so they can be analysed in detail. The above models are an extension of the model of STN-GPe circuit considered previously [11, 32, 33], differing in that it includes an additional excitatory—inhibitory oscillator for the cortex. The structure of the models is shown in Fig 1C and 1D. In both models the STN neural population projects excitatory glutamatergic axons to the GPe, and the GPe neural population projects inhibitory GABAergic axons back to the STN as well as to other neurons within the GPe. In addition, the cortex is represented by a similar circuit of excitatory and inhibitory populations with reciprocal projections. In both models cortex projects to STN.

Additionally in the feedback model (Fig 1D) a closed loop is formed by inhibitory projections from the STN back to the cortex. To achieve the simplicity of the model, we followed the approach of Dovzhenok and Rubchinsky [35] and instead of explicitly modelling the activity in all neural populations in the feedback loop connecting the STN to the cortex (indicated by dashed lines in Fig 1A), we captured it in a single connection with time delay. Since the projections between the STN-GPi-thalamus-cortex, contains two excitatory projections and one inhibitory projection (dashed line in Fig 1A), the overall action of the time delayed projection is inhibitory (as indicated in Fig 1C). However, the thalamic feedback to the STN is excitatory, hence the input to the cortex in the model includes a constant positive component, which makes it positive overall (indicated by *C* in Fig 1C).

We do not incorporate a feedback loop via the indirect pathway (cortex-striatum-GPe) because experimental evidence suggests that it is not critical for beta oscillation generation (Figure 10 in Tachibana et al. [14]). These authors show that, in a monkey model of Parkinson’s disease, when the striatal inputs to the recorded GPe neurons are blocked by gabazine microinjections, the power of beta oscillations in the GPe is not reduced. The only effect of this manipulation is a modest increase in the mean firing rate of GPe neurons. Thus in our model the GPe receives a constant inhibitory input from the striatum.

Mathematically, the models are given by:

$$\begin{array}{cc} \tau_{S}S^{\prime } & =F_{\text{S}}\left(w_{\text{CS}}E\left(t-T_{CS}\right)-w_{\text{GS}}G\left(t-T_{GS}\right)\right)-S\left(t\right)\\ \tau_{G}G^{\prime } & =F_{\text{G}}\left(w_{\text{SG}}S\left(t-T_{SG}\right)-w_{\text{GG}}G\left(t-T_{GG}\right)-Str\right)-G\left(t\right)\\ \tau_{E}E^{\prime } & =F_{\text{E}}\left(-w_{\text{SC}}S\left(t-T_{SC}\right)-w_{\text{CC}}I\left(t-T_{CC}\right)+C\right)-E\left(t\right)\\ \tau_{I}I^{\prime } & =F_{\text{I}}\left(w_{\text{CC}}E\left(t-T_{CC}\right)\right)-I\left(t\right) \end{array}$$ (1)

In Eq 1, *S*(*t*), *G*(*t*), *E*(*t*) and *I*(*t*) are the firing rates of the STN, GPe, excitatory and inhibitory populations respectively. Similarly, *S*′(*t*), *G*′(*t*), *E*′(*t*) and *I*′(*t*) are the derivatives of the firing rates of the STN, GPe, excitatory and inhibitory populations respectively. The parameters *T*_*ij*_ represent the time delays of synaptic connections between neural populations. The subscript *i* indicates the population from which the signal originates and the subscript *j* indicates where the signal is received. Hence *T*_*GS*_ describes the time delay from the GPe population to the STN population, *T*_*SG*_ is the time delay between the STN and GPe populations, *T*_*GG*_ is the time delay of the self-inhibitory connection of the GPe population, *T*_*CS*_ is the time delay from the excitatory population of the cortex to the STN and *T*_*SC*_ is the time delay of the STN to the excitatory population of the cortex. The parameters *w*_*ij*_ represent the synaptic weights of these connections, with the same labelling convention for the subscripts *i* and *j* as for the time delays. The weights reflect the impact of a change in firing rate of presynaptic neurons on the firing rate of postsynaptic neurons (see Nevado-Holgado et al. [32] for details). Thus a high value of weight in a model may represents high strength of connections, or a large number of connections, or a relatively large number of neurons in one area projecting to a relatively small area, or their combinations. In the resonance model, the parameter *w*_*SC*_ is forced to 0, while in the feedback model, it can take any non-negative value.

The membrane time constants for the STN, GPe, E and I populations are denoted by *τ*_*S*_, *τ*_*G*_, *τ*_*E*_ and *τ*_*I*_ respectively. A constant input is provided to the excitatory population of the cortex, denoted by *C*, to represent a constant component of extrinsic and intrinsic excitatory inputs (see above), and *Str* to the GPe that represents the constant inhibitory input from striatum. Finally, the terms *F*_*S*_(*in*), *F*_*G*_(*in*), *F*_*E*_(*in*) and *F*_*I*_(*in*) are the activation functions of the STN, GPe, E and I neural populations respectively, given by Eq 2. These functions describe the population firing rate as a function of synaptic input, *in*

$$\begin{array}{cc} F_{\text{S}}\left(in\right) & =\frac{M_{\text{S}}}{1+\left(\frac{M_{\text{S}}-B_{\text{S}}}{B_{\text{S}}}\right)exp\left(\frac{-4in}{M_{\text{S}}}\right)}\\ F_{\text{G}}\left(in\right) & =\frac{M_{\text{G}}}{1+\left(\frac{M_{\text{G}}-B_{\text{G}}}{B_{\text{G}}}\right)exp\left(\frac{-4in}{M_{\text{G}}}\right)}\\ F_{\text{E}}\left(in\right) & =\frac{M_{\text{E}}}{1+\left(\frac{M_{\text{E}}-B_{\text{E}}}{B_{\text{E}}}\right)exp\left(\frac{-4in}{M_{\text{E}}}\right)}\\ F_{\text{I}}\left(in\right) & =\frac{M_{\text{I}}}{1+\left(\frac{M_{\text{I}}-B_{\text{I}}}{B_{\text{I}}}\right)exp\left(\frac{-4in}{M_{\text{I}}}\right)} \end{array}$$ (2)

where the constants *M*_*S*_, *M*_*G*_, *M*_*E*_ and *M*_*I*_ are the maximum firing rates of each population, and *B*_*S*_, *B*_*G*_, *B*_*E*_ and *B*_*I*_ are the population firing rates in the absence of input. The other two parameters needed to define a sigmoid curve are the minimum firing rate, which is set to zero (as often seen in experiments), and the slope, which is set to 1 to allow an interpretation of synaptic weight units [32].

### The parameters of the model

The values of all parameters, except for connections weights (*w*_*ij*_), were estimated directly on the basis of experimental data, briefly reviewed in this section. In the next section details are provided of how the weights were estimated on the basis of the recent data set provided by Tachibana et al. [14].

As in previous work by Nevado-Holgado et al. [32], the parameters of the model were based on data from experiments on human patients, non-human primates (which are evolutionary close to humans), or rats if no primate data were available or suitable. The parameters used are listed in Table 1. Many parameters have the same value as in the previous work [32] and their choice has been justified in detail in that work. For parameters used for the cortical populations a realistic range of values were chosen. Below we review data on the basis of which we estimated parameters that have different values than in the previous work [32].

**Table 1:** The parameters and their values used together with the sources for this information.

Parameter	Parameter value	Source
*T*_*SG*_ & *T*_*GS*_	6*ms*	Nevado-Holgado et al. [32] use the same time delay for both the STN to GPe and GPe to STN connections based on findings by Fujimoto and Kita [62] and Kita et al. [63]
*T*_*GG*_	4*ms*	Based on proximity between cells [32]
*T*_*CS*_	5.5*ms*	Average of range reported by Devergnas and Wichmann [56]
*T*_*SC*_	21.5*ms*	Average of range reported by Devergnas and Wichmann [56]
*T*_*CC*_	1–10*ms*	Wide range of values allowed
*τ*_*S*_	12.8*ms*	From Gillies and Willshaw [53]
*τ*_*G*_	20*ms*	Reinterpretation of data in [61]
*τ*_*E*_	10–20*ms*	Due to heterogeneity of data available, values were chosen that could represent a range of published values of membrane time constants [64–66] (in rats), [67] (in mice)
*τ*_*I*_	10–20*ms*	Due to heterogeneity of data available, values were chosen that could represent a range of published values of membrane time constants [68]
*M*_*S*_	300*spk*/*s*	Hallworth et al. [69]
*B*_*S*_	10*spk*/*s*	Abbott et al. [70] report a value of 10.1*spk*/*s* and Baufreton et al. [71] report 7.8 ± 2.6*spk*/*s*
*M*_*G*_	400*spk*/*s*	Kita et al. [63] and Kita [72]
*B*_*G*_	20*spk*/*s*	*In vitro* recordings show, in the absence of all synaptic inputs, that GPe neurons fire rhythmically at rates between 10 and 20 Hz [28]
*M*_*E*_	50–80*spk*/*s*	[73]
*B*_*E*_	0–20*spk*/*s*	[73, 74]
*M*_*I*_	200–330*spk*/*s*	[73]
*B*_*I*_	0–20*spk*/*s*	[73]

The delay time of the long loop arises due to the sum of delay times of two components. These include the direct connection between cortex and STN, denoted by *T*_*CS*_ and by the pathway from STN to cortex, which is formed by the pathways connecting STN-thalamus-GPi-cortex and is denoted by *T*_*SC*_. Therefore, the closed loop forms the following pathway: STN-GPi-thalamus-cortex-STN. Meta-analysis of human Parkinson’s patients [56] reports time delays from STN to cortex in the range of 3–8*ms* and time delays from STN to cortex in the range of 18–25*ms*. We chose the values as the mean of these ranges so that *T*_*SC*_ = 5.5*ms* and *T*_*SC*_ = 21.5*ms*. It is difficult to constrain the delay within the cortex *T*_*CC*_, because the cortical circuit involved in generating beta oscillations may involve multiple neural populations, while we modelled just two of them, so we left *T*_*CC*_ as a free parameter of the model and constrain its value between 1–10*ms*.

We wished to choose the values of the parameters describing neuronal firing rate in the absence of input *B*_*S*_ and *B*_*G*_ on the basis of the same species. The values used by Nevado-Holgado et al. [32] were based on different species and consequently were very dissimilar, which was found to cause an imbalance in the relationship between the STN and GPe populations that required an unrealistically strong striatal input to reproduce experimental data. Since there is no data available on the value of *B*_*S*_ in primates, we used the estimates from rats for both *B*_*S*_ and *B*_*G*_.

Because of the heterogeneity of cortical neuronal types and their associated properties in the cortex and also because of inadequate knowledge about which neurons contribute to beta oscillations in cortex we chose a range of values from the parameters *B*_*E*_, *B*_*I*_, *M*_*S*_ and *M*_*G*_. For *B*_*E*_ and *B*_*I*_ the range used was between 0–20*spk*/*s*. For *M*_*E*_ the range used was 50–80*spk*/*s*, while for *M*_*I*_ the range was 200–330*spk*/*s*.

For the STN membrane time constant we used *τ*_*S*_ = 12.8*ms*, which is reported in the study of Gillies and Willshaw [53]. Gillies and Willshaw [53] suggest that the reason for why Kita et al. [57] measured the STN constant to be only *τ*_*S*_ = 6*ms* is that penetration of the soma with a sharp electrode caused a decrease in the soma resistance leading to a decreased soma time constant and faster depolarisation of the cell [58, 59]. The alternative method, using a patch or whole cell recording, provides more faithful representation of the neural dynamics [60].

**Fig 7:**
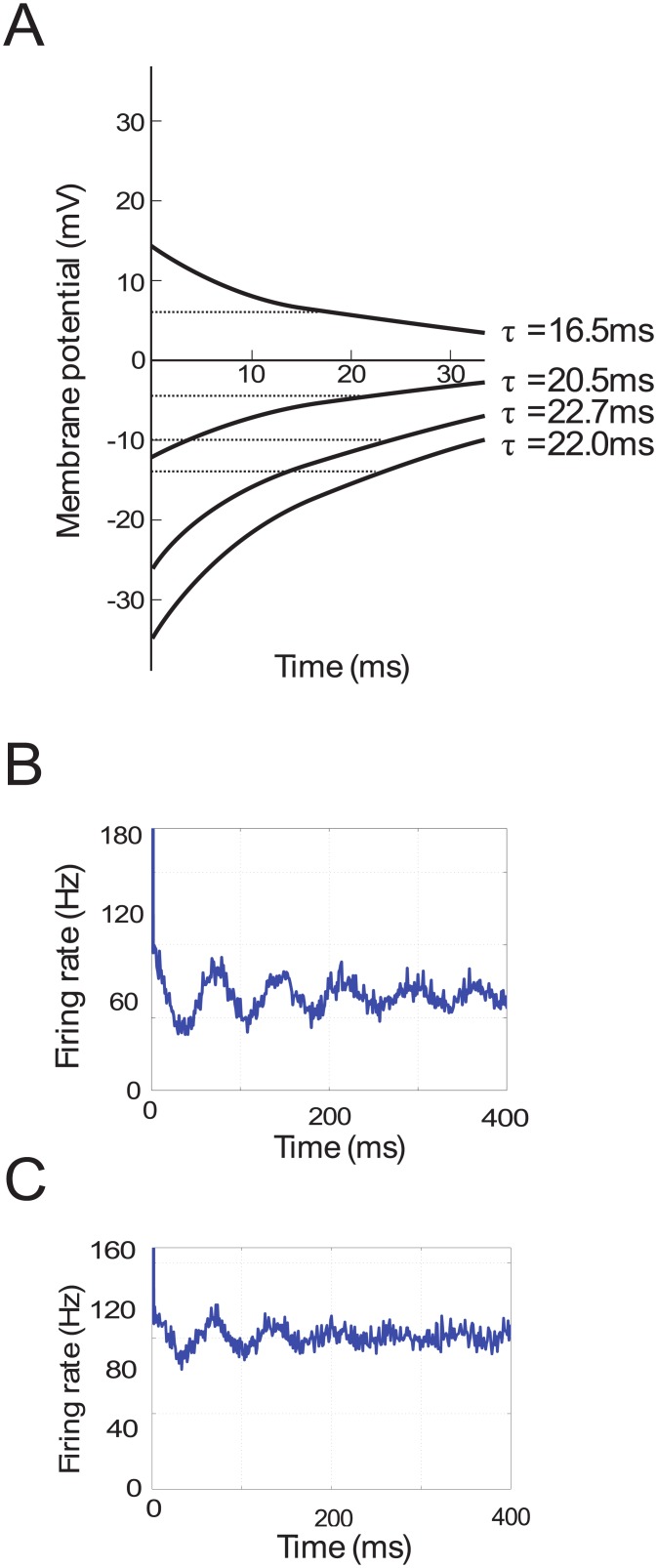
Experimental data used to estimate parameters of the models. A) Changes in the membrane potential of a GPe neuron following current injections. Data taken from Kita and Kitai [61] (Fig 2, Panel A). Dashed lines indicate *V*_0_ *e* ^−1^. B, C) Simulated auto-correlograms of STN and GPe neurons.

We are not aware of explicit reports of the value of the time constant of the GPe, but here we more precisely estimated this time constant from the data presented in Fig 7A, modified from Kita and Kitai [61]. Namely, we used the information about the rate at which the membrane potential recovers in order to calculate the membrane time constants. When the membrane potential is falling, the dependence on time is described by

$$V_{m}\left(t\right)=V_{0}e^{-t/\tau }$$ (3)

where *V*_*m*_ is the membrane potential and *V*_0_ is the resting potential of the neuron. Note that after a period of one time constant (i.e. when *t* = *τ*) the function reaches *V*_0_ *e* ^−1^, therefore the membrane time constant can be calculated as the time it takes for the membrane potential to reach *V*_0_ *e* ^−1^ as illustrated in Fig 7A. The membrane time constants can be read from the graph at the points where the rising membrane potential has recovered approximately 0.63 (i.e. 1−*e* ^−1^) towards the resting potential, and the falling membrane potential has reduced to approximately 0.37 (i.e. *e* ^−1^) of the resting potential. The average of the four *τ* values indicated in the figure is approximately 20*ms*. The remaining two curves were not taken into consideration because it was difficult to get a clear measurement. In any case, although this value is approximate, a small variation in the membrane time constant has only a small effect on frequency of the model.

As for the other parameters of the cortical populations the membrane time constant *τ*_*E*_ for excitatory neurons and *τ*_*I*_ for inhibitory neurons were set within a realistic range of 10–20*ms*.

### Fitting the models to experimental data

We wanted to find values of *w*_*ij*_ which could reproduce patterns of activity in a monkey model of Parkinson’s disease and the effects of blocking different connections, as observed by Tachibana et al. [14]. Different STN and GPe neurons recorded by Tachibana et al. [14] had different mean firing rates and amplitude of oscillations, but unfortunately the amplitudes of oscillations were not reported for all neurons (or even an average), and they could only be inferred from figures for single neurons. In this paper we present the simulations of the model fitted to data from individual neurons reported by Tachibana et al. [14]. An alternative method was considered, which estimated the average mean firing rates and amplitudes of neurons recorded by Tachibana et al. [14]. In order to get a value for the mean amplitudes the assumption was made that all oscillating neurons have maximum amplitude so that the average amplitude over all neurons could be calculated by multiplying the percentage of oscillatory cells recorded by Tachibana et al. [14] with the mean firing rates of all neurons. These results are not included in this paper but were presented by Pavlides [75], and were similar to those obtained by fitting data from individual neurons.

We wished to show that the models could reproduce the mean firing rate, frequency and amplitude of beta oscillations observed in individual STN and GPe neurons. These properties of activity can be inferred from auto-correlograms recorded from the STN and GPe neurons (Fig 7B and 9H in [14]). In order to estimate the firing rates and oscillation amplitudes from these auto-correlograms, it was necessary to reverse engineer how they could arise. A noisy sinusoidal oscillation in the firing rate was constructed and used to generate spikes from an inhomogeneous Poisson process. More specifically, the equation of the firing rate is given by

fr(t)=Asin(θ)+b (4)

dθ=2πfdt+Ndw (5)

where *fr*(*t*) is the firing rate as a function of time, *A* is the amplitude and *b* is the baseline firing rate. The phase *θ* is given by the Eq 5, in which the phase advances on average according to the frequency *f*, but is subject to noise described by a Wiener process, where *N* is a constant that modifies the strength of the noise, *dt* is a time step and *dw* is a random number from a Gaussian distribution with zero mean and variance *dt*.

While generating synthetic spike trains (of length 40*s*), the total time was divided into short time bins of *dt* = 1*ms* and each of these time bins contained either 1 spike with probability *fr*(*t*)*dt* or 0 spikes. Auto-correlograms were then constructed from these spike trains. We looked for parameters *A*, *b*, *f* and *N* for which the auto-correlogram from Fig 7B and 9H in Tachibana et al. [14] best matched the simulated auto-correlograms. These are shown in Fig 7B and 7C.

For the following parameters, the generated auto-correlograms matched those from Fig 7B and 9H in Tachibana et al. [14]. For the STN, *b* = 65*spk*/*s*, *A* = 60*spk*/*s*, *f* = 13.7Hz and *N* = 3.3 whereas for the GPe, *b* = 100*spk*/*s*, *A* = 55*spk*/*s*, *f* = 14.6Hz and *N* = 3.9. Using these values the mean (*b*), minimum (*b*−*A*) and maximum (*b* + *A*) firing rates were calculated (Table 2) and these values were used as constraints in the model fitting.

**Table 2:** Summary of experimental data used to constrain the model. This includes the minimum, mean and maximum firing rates of the STN and GPe populations and the frequency of pathological oscillation.

Firing Rates	Firing rate value
Minimum STN	5 spk/s
Mean STN	65 spk/s
Maximum STN	125 spk/s
Minimum GPe	45 spk/s
Mean GPe	100 spk/s
Maximum GPe	155 spk/s
Frequency	14 Hz

We also wished the model to reproduce the observations of Tachibana et al. [14], which showed that excessive beta oscillations can be stopped by blocking connections *STN* → *GPe*, *GPe* → *STN* or *Ctx* → *STN* but not by blocking connections *Str* → *GPe*. Thus we searched for the values of the connection weights *w*_*ij*_ for which the oscillations with characteristics as in Table 2 were present, and were then suppressed when we set *w*_*SG*_ = 0 or *w*_*GS*_ = 0 or *w*_*CS*_ = 0, but were not suppressed by setting *Str* = 0.

When blocking *w*_*CS*_ the model could cease oscillating simply due to a lack of excitability [32]. However, Tachibana et al. [14] did not observe a change in the STN mean activity after blocking the *Ctx* → *STN* connection, so the change of excitability was not the reason for the disappearance of oscillations in their experiment. Therefore, we investigated if the oscillations can be stopped in STN after blocking the connections between cortex to STN without changing its level of excitability. Specifically, to ensure that the activity of the STN remains constant after setting *w*_*CS*_ = 0, an additional input *C*_*adj*_ applied to the STN which is equal to the average activity *E* of excitatory cortical population prior to blockade, multiplied by the connection weight *w*_*CS*_.

Approximate connection weights were found manually which were then used as starting points for a simulated annealing optimization algorithm (*simulannealbnd* function in MATLAB). In total 15 parameters were optimized. There were 8 free parameters: *w*_*SG*_, *w*_*GS*_, *w*_*CS*_, *w*_*SC*_, *w*_*GG*_, *w*_*CC*_, *C* and *Str* that could take any values in a wide range (0–10 for weights, and 0–30 for *C* and *Str*). We also fitted 7 other parameters: *T*_*CC*_, *τ*_*E*_, *τ*_*I*_, *B*_*E*_, *B*_*I*_, *M*_*E*_ and *M*_*I*_ which were constrained to ranges given in Table 1. For each set of parameters, the optimization algorithm solves Eq 1 numerically, using the DDE23 function in MATLAB, and various oscillation measures are calculated, such as the minimum, mean and maximum firing rates and oscillation frequency. These simulated data are compared with experimental data using a cost function, given by

$$\begin{array}{ccc} Cost & = & \sum_{i\in \{S,G\}}{\left(Mn_{D,i}-Mn_{M,i}\right)}^{2}+{\left(Av_{D,i}-Av_{M,i}\right)}^{2}+{\left(Mx_{D,i}-Mx_{M,i}\right)}^{2}\\ & + & k*{\left(Freq_{D}-Freq_{M}\right)}^{2}\\ & + & \sum_{cut=1}^{3}\sum_{i\in \{S,G\}}{\left(Mx_{M,i}^{cut}-Mn_{M,i}^{cut}\right)}^{2}\\ & + & \sum_{i\in \{S,G\}}{\left.[\left(Mx_{D,i}-Mn_{D,i}\right)-\left(Mx_{M,i}^{cut}-Mn_{M,i}^{cut}\right)\right]}^{2} \end{array}$$ (6)

The above cost function includes multiple terms which we will now explain. The first line compares the Minimum, Average and Maximum firing rates within the oscillation cycle for the intact network, which we denote by *Mn*, *Av* and *Mx*, respectively. The indices *D* and *M* denote Data (from Table 2) and Model simulations, respectively. The summation index *i* ranges between the firing rates for the STN and GPe populations. The second line compares the frequency, which is the same for both populations in the model. An additional weight, *k* = 20, was given to the frequency to emphasize its importance in the optimisation.

The third line is used to ensure that the amplitude of oscillations in the model decreases towards 0 after blocking connections: STN to GPe, GPe to STN and Ctx to STN (indexed by *cut*). This amplitude should decrease in both STN and GPe (indexed by *i*).

The final line of the cost function is included to represent the effect of blockade of striatal input to the recorded GPe neurons. For this the amplitude of oscillations in both the STN and GPe should not decrease, therefore the constraint is that the amplitude of oscillations in the model must not be lower after blockade than the amplitude of oscillations given by the difference between the maximum and minimum firing rates given in Table 2. It was also shown by Tachibana et al. [14] that while the power of beta oscillations is unchanged, a lesion of striatal input also slightly increases the mean firing rate of the GPe population. This was not included as a constraint in the cost function but it is seen in the results of the fitting. Additionally, a strong penalty for parameters outside allowed ranges was also imposed in the cost function as a final constraint (not shown).

The Matlab code for the model is available in the repository ModelDB with accession number 184491 (http://modeldb.yale.edu/184491).

### Analysing the effect of delay on frequency

While investigating the effects of delays in the long loop on the frequency of the oscillations, we simulated the feedback model for the parameters corresponding to Fig 3C, except for parameters *T*_*CS*_ and *T*_*SC*_ which we varied systematically. In particular, in each simulation we scaled both *T*_*CS*_ and *T*_*SC*_ by a constant *α*, that was different in different simulations. The horizontal axis in Fig 6A shows the total delay defined as *T*_*SS*_ = *αT*_*CS*_ + *αT*_*SC*_.

In order to obtain an analytic condition for the stability of the model, it needs to be simplified as shown in Fig 1E, such that it is linear and only describes the time delays in the STN-GPi-thalamus-cortex-STN loop. We set *F*_*S*_(*x*) = *x* and *F*_*G*_(*x*) = *x*. In the stability analysis the constant extrinsic and intrinsic excitatory inputs to STN and the striatal inputs, *C* and *Str*, do not affect stability because they can be scaled away. This occurs because if *S*(*t*) = *s*(*t*) + *s*_0_ and *G*(*t*) = *g*(*t*) + *g*_0_, it is always possible to choose *s*_0_, *g*_0_ so that *s*(*t*), *g*(*t*) are the solution of the model with no *C* or *Str* terms. In addition, the parameter *w*_*GG*_ > 0 makes the system more stable (because it behaves as a leak in the system), but it does not qualitatively change the dynamics of the stability boundary, therefore we also set this to zero. Finally, we rescale time by setting *τ* = 1 so that the time delay *T* becomes $\frac{T}{\tau }$. The resulting equations take the form

$$\begin{array}{cc} s^{\prime }\left(t\right) & =-w_{GS}g\left(t\right)-w_{SS}s\left(t-T_{SS}\right)-s\left(t\right)\\ g^{\prime }\left(t\right) & =w_{SG}s\left(t\right)-g\left(t\right) \end{array}$$ (7)

Eq 7 are equivalent to a forced damped harmonic oscillator with time delayed restoring force. The stability analysis of the system was performed by Cooke and Grossman [36]. To clarify how the results of Cooke and Grossman [36] can be applied to our model, we rewrite the pair of Eq 7 as a single second order delay differential equation.

$$s^{\prime \prime }\left(t\right)+2s^{\prime }\left(t\right)+\left(w_{\text{GS}}w_{\text{SG}}+1\right)s\left(t\right)+w_{\text{SS}}s\left(t-T\right)+w_{\text{SS}}s^{\prime }\left(t-T\right)=0$$ (8)

The above equation has the same form as Equation 11 in Cooke and Grossman [36], and comparing the two shows the relationship between the parameters in the two papers. The stability boundary given in Equation 15 of Cooke and Grossman [36] is plotted in Fig 6A, and compared with the results of the simulations of the linear model. The time axis has been rescaled using a membrane time constant of *τ* = 16*ms*, which is the rounded average of those used in the full model.
